# Impact and Sustainability of Antibiotic Stewardship on Antibiotic Prescribing in Visceral Surgery

**DOI:** 10.3390/antibiotics10121518

**Published:** 2021-12-11

**Authors:** Magdalena Monika Gruber, Alexandra Weber, Jette Jung, Jens Werner, Rika Draenert

**Affiliations:** 1 Antibiotic Stewardship Team, University Hospital, LMU Munich, 81377 München, Germany; magdalena.gruber@med.uni-muenchen.de (M.M.G.); alexandra.weber@med.uni-muenchen.de (A.W.); jette.jung@med.uni-muenchen.de (J.J.); 2 Hospital Pharmacy, University Hospital, LMU Munich, 81377 München, Germany; 3 Max von Pettenkofer Institute, Faculty of Medicine, LMU Munich, 81377 München, Germany; 4 Department of General, Visceral und Transplantation Surgery, University Hospital, LMU Munich, 81377 München, Germany; jens.werner@med.uni-muenchen.de

**Keywords:** antibiotic stewardship, visceral surgery, sustainability, antibiotic consumption

## Abstract

Background: Antibiotic stewardship (AS) ward rounds are a core element in clinical care for surgical patients. Therefore, we aimed to analyze the impact of surgical AS ward rounds on antibiotic prescribing, and the sustainability of the effect after the AS interventions are no longer provided. Methods: On four wards of the department of visceral surgery, we conducted two independent retrospective prescribing analyses (P1, P2) over three months each. During the study periods, the level of AS intervention differed for two of the four wards (ward rounds/no ward rounds). Results: AS ward rounds were associated with a decrease in overall antibiotic consumption (91.1 days of therapy (DOT)/100 patient days (PD) (P1), 70.4 DOT/100PD (P2)), and improved de-escalation rates of antibiotic therapy (W1/2: 25.7% (P1), 40.0% (P2), *p* = 0.030; W3: 15.4 (P1), 24.2 (P2), *p* = 0.081). On the ward where AS measures were no longer provided, overall antibiotic usage remained stable (71.3 DOT/100PD (P1), 74.4 DOT/100PD (P2)), showing the sustainability of AS measures. However, the application of last-resort compounds increased from 6.4 DOT/100PD to 12.1 DOT/100PD (oxazolidinones) and from 10.8 DOT/100PD to 13.2 DOT/100PD (carbapenems). Conclusions: Antibiotic consumption can be reduced without negatively affecting patient outcomes. However, achieving lasting positive changes in antibiotic prescribing habits remains a challenge.

## 1. Introduction

Antibiotic resistance is a major global threat, making effective antibiotic treatment increasingly difficult, while the process of developing new antibiotics still has room for improvement [1]. Broad use of, in particular, last-resort compounds, such as oxazolidinones or carbapenems, is a serious concern worldwide, as antibiotic mis- and overuse in health care are known to be main drivers of antibiotic resistance [2,3]. Rates of up to 47% of inappropriate antibiotic prescribing have been found in surgical specialties [4,5,6,7]. To promote judicious anti-infective therapy, infectious disease societies are demanding the implementation of antimicrobial stewardship programs (ASPs), as infections caused by multidrug-resistant bacteria result in increased mortality, a prolonged hospital stay, and higher health care costs [8].

In visceral surgery, intra-abdominal infections (IAI) are associated with high morbidity and mortality. Antibiotics, in addition to surgical source control, are indispensable in their treatment [9]. However, optimal antibiotic therapy in IAI remains a challenge. Interestingly, most ASPs in surgery focus on the perioperative setting (i.e., antibiotic prophylaxis) and management of surgical site infections, and neglect pre- and postoperative antibiotic use [10]. Since Charani et al. highlighted the importance of surgical ward rounds for the clinical care of patients [11], our aim was to evaluate the immediate and longer-term impacts of antibiotic stewardship (AS) ward rounds on antibiotic prescribing in a visceral surgery department. Therefore, AS ward rounds were provided on three wards of a visceral surgery department where a steady increase in antibiotic consumption was observed over the previous years.

ASPs can effectively reduce inappropriate antibiotic consumption without compromising patient outcomes, but the sustainability of AS interventions has not been studied in detail to date [12]. AS activities have to be prioritized to areas with the greatest need. So, ASPs often face limited staff and time resources and, therefore, have difficulties being permanently present. Thus, the present study also investigated if the effects of AS ward rounds were sustainable and sufficiently maintained, even after AS ward rounds were no longer provided to that area.

## 2. Results

### 2.1. Patient Characteristics

**Figure 1:**
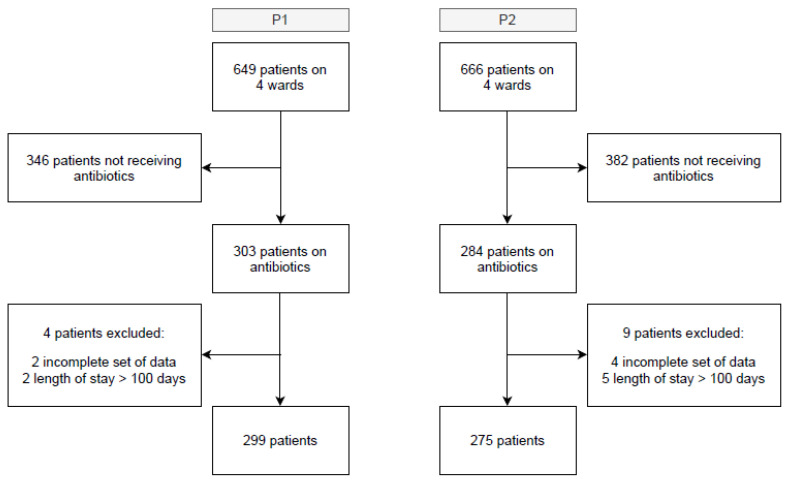
Flow chart of all patients admitted to four wards of the visceral surgery department during P1 and P2.

Throughout the two study periods (P1, P2), a total of 649 (P1) and 666 (P2) patients were admitted to the four surgical wards. Of those, 46.7% (303, P1) and 42.6% (284, P2, *p* = 0.14) received at least one course of systemic antibiotic treatment during their hospital stay. During P1, four patients were excluded from the study, due to incomplete sets of data (two patients) and lengths of stay longer than 100 days (two patients), whereas nine patients were excluded in P2, due to incomplete sets of data (four patients) and lengths of stay of over 100 days (five patients). The statistical analysis included 299 (P1) and 275 (P2) patients (Figure 1). The median age was 62 years in both groups. In P2, more women were included than in P1 (36.8% (P1), 45.8% (P2), *p* = 0.028). The Charlson comorbidity index [13] and the allocation of diagnoses to the different organ systems were comparable in both study cohorts. Surgery was performed in 212 (70.9%) and 213 (77.5%, *p* = 0.074) patients in P1 and P2, respectively. Rates of multidrug-resistant bacteria isolated (methicillin-resistant Staphylococcus aureus, vancomycin-resistant enterococci, linezolid-resistant Staphylococcus epidermidis, extended-spectrum β-lactamase producing gram-negative bacteria, and carbapenemase-producing Enterobacteriaceae) were similar in the two study periods. Results of nasopharyngeal, inguinal, or rectal swabs for screening of colonization were excepted. There were no differences in median length of stay, readmission for infection after 30 days, or in-hospital mortality between the two study cohorts (Table 1).

### 2.2. Changes in Overall Antibiotic Use and Choice of Substance

**Figure 2:**
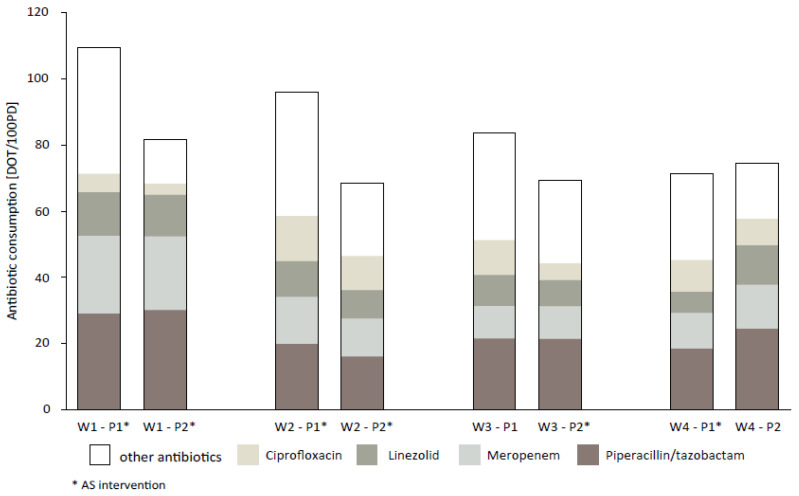
Overall antibiotic consumption, and consumption of ciprofloxacin, linezolid, meropenem, and piperacillin/tazobactam on the four wards, comparing P1 and P2.

A decrease in overall antibiotic consumption was observed in P2 on ward 1 (W1) and ward 2 (W2), where AS ward rounds were conducted in both phases, and on ward 3 (W3), where AS ward rounds took place only in P2 (W1/2/3 combined: 91.1 days of therapy (DOT)/100 patient days (PD) vs. 70.4 DOT/100PD). On ward 4 (W4), the AS ward rounds were no longer provided during P2. The overall antibiotic consumption remained unchanged on this ward. On all four wards, reduced usage of cefuroxime, ciprofloxacin, and metronidazole prescriptions was seen during study period P2 (cefuroxime: 3.1 DOT/100PD vs. 1.1 DOT/100PD, ciprofloxacin: 10.5 DOT/100PD vs. 7.1 DOT/100PD, metronidazole: 10.7 DOT/100PD vs. 5.2 DOT/100PD; Figure 2). Additional data on antibiotic consumption for individual substances according to the respective wards are available in the Supplementary Materials (Table S1).

In conclusion, while AS ward rounds were actively performed, we observed an improvement in antibiotic use on the wards. Looking at the overall antibiotic consumption and, in particular, the application of cephalosporins, fluoroquinolones, and metronidazole, the reductions in broad-spectrum and restricted antibiotics were less pronounced.

### 2.3. Length of Therapy and Prescribing Behavior

Shortening the duration of antibiotic therapy, where possible, without negatively influencing therapy safety is an important goal of AS. However, in our study, the length of antibiotic therapy was a median of 8 days in both study periods, and provision of AS ward rounds did not have an impact on length of therapy. The total number of prescriptions was 342 courses of antibiotics (COAs) in phase 1, and 312 COAs in phase 2. Initial doses were given intravenously in 88.9% (P1) and 90.7% (P2), respectively. No effect of the AS ward rounds on the intravenous-to-oral (iv-to-oral) switch rate nor on the duration until iv-to-oral-switch could be detected.

On W1 and W2, the antibiotic therapy was de-escalated more often in P2 than in P1 (25.7% vs. 40.0%, *p* = 0.030). On W3, a trend of increased de-escalation rate during a COA was observed in P2 (*p* = 0.081). On W4, there was a tendency towards a decrease of iv-to-oral switch and de-escalation rates; however, it was not statistically significant. Duration to de-escalation did not change significantly on the four wards (Table 2).

Therefore, AS ward rounds were associated with increased de-escalation rates, but did not reduce the duration of antibiotic therapy.

## 3. Discussion

This retrospective monocentric study investigated the impact of AS ward rounds on antibiotic prescribing behavior in a visceral surgery department, as well as the sustained effects after the AS ward rounds were stopped. To analyze the change in antibiotic prescribing patterns, depending on the involvement of performed AS measures, the study was conducted on four surgical wards with different statuses in implementation of AS ward rounds. Antibiotic consumption decreased, while de-escalation rates increased, when AS measures were implemented on the wards. AS ward rounds were associated with improved antibiotic prescribing, and had sustained effects regarding overall antibiotic consumption for the following three months after they were no longer provided. This is corroborated by the fact that overall antibiotic consumption steadily increased on the four wards over the years before the study was conducted, and changed after AS ward rounds were provided on the wards. Furthermore, the four wards had comparable collectives of patients with visceral surgical foci, and the diagnoses the patients were admitted for, or the disease severity (Charlson comorbidity index), did not change over time. In addition, there was no change of personnel in crucial positions. The decrease in overall antibiotic consumption on W1 might be explained by reductions in inappropriate COAs, as there was no reduction in length of antibiotic therapy in P2. In particular, reduced use of the last-resort compounds linezolid and meropenem on W2 and W3 is noteworthy, as it was not compensated by the use of other broad-spectrum antibiotics, such as piperacillin/tazobactam.

Previous studies observed a positive impact of AS in surgical patients with IAI [14,15,16]. They showed improved antibiotic use after the implementation of an ASP, based on the development of guidelines regarding antibiotic therapy of IAI. Our study also highlights the impact of AS ward rounds in improving antibiotic prescribing. Our results are in accordance with the ones previously described by Surat et al. [17]. In addition, a reduction in total days of antibiotic therapy was observed by implementing an ASP, including regular ward rounds, but without local standards for antibiotic therapy of IAI at that time. In general, it seems to be difficult to achieve shorter durations of antibiotic therapy, even though a reduction in length of therapy is widely recommended in the literature for IAI with adequate source control [18,19,20,21,22]. Except for Surat and colleagues, none of the previous studies could show a reduction of antibiotic therapy duration, and the shortened duration of therapy was limited to the treatment of cholecystitis—a disease that was strongly underrepresented in our cohort.

The positive effects of performed AS ward rounds regarding antibiotic consumption in general were sustained, but not for last-resort compounds like carbapenems or oxazolidinones. On W4, where AS ward rounds were stopped after P1, the overall antibiotic consumption remained on a stable level, but the use of meropenem and linezolid distinctly increased in P2. Barbieri et al. investigated the sustainability of clinical pathways for decreasing use of broad-spectrum antibiotics in pediatric patients suffering from acute otitis media or pharyngitis [23]. A lack in sustained effect of the implemented clinical pathways after ending their educational support was observed, consistent with our findings. Ullman and colleagues investigated the lasting economic impact of an ASP, after stopping and restarting [24]. The results support our findings of recommending a continuation of the ASP, although the evaluated parameter of antibiotic purchases is not the suitable metric to use. However, at the start of this ASP, the currently recommended metrics (defined daily doses and days of antibiotic therapy) had not been established yet. In contrast, Dona and colleagues’ AS intervention focusing on improved perioperative antibiotic prophylaxis, resulted in lasting effects 24 months after ending the educational lectures for their clinical pathways [25]. However, pre- and postoperative antibiotic therapies appear more challenging than surgical antibiotic prophylaxis, in the majority of cases.

Charani et al. investigated antibiotic decision making in surgery and pointed out the importance, but also the difficulties, of ward rounds for patient care in surgery [11,26]. Our findings support their observations. Our experience was that junior faculty were present on the AS ward rounds, while the senior faculty were in the operating room (OR) and were, therefore, difficult to reach. Face-to-face communication was further complicated, due to their limited time resources on the wards. Many duties outside the OR were delegated to junior surgeons, and discussions about antibiotic therapy during AS ward rounds were, therefore, mostly conducted with them. Recommendations regarding antibiotic therapy made during AS ward rounds often had to be approved later by the senior faculty. Thus, final decisions about antibiotic therapy were made after the AS ward rounds and without the involvement of the AS team. When a junior doctor reports to the senior faculty, there could be a loss of information regarding the recommendations, which could lead to rejection of those AS recommendations. However, the junior faculty that we dealt with was mostly interested in the help by the AS team. An open mind for AS measures is an important prerequisite for changing habits. Charani and colleagues also described surgeons as being afraid of negative patient outcomes, which could be a driver for inappropriate antibiotic use, especially postoperatively—a fact that we can confirm with our experience.

In addition to the AS ward rounds, the strong decrease of ciprofloxacin consumption on all four wards might have been influenced by the publication of an official drug-safety warning regarding the restricted application of fluoroquinolones after P1 (10/2018). This topic has been widely discussed in the media and has been a focus of the public’s attention. Due to the low bioavailability of cefuroxime, it was excluded from the institution’s formulary. This could have additionally reduced cefuroxime consumption, as well as causing a reduction in prolonged perioperative antibiotic prophylaxis with cefuroxime. The department’s internal guidelines for perioperative antibiotic prophylaxis were reviewed and reissued between the two study periods, and explicitly suggested single-shot application, but no prolonged postoperative therapy regime. Consequently, the consumption of metronidazole, co-administered with cefuroxime and ciprofloxacin, decreased on the wards to the same extent as cefuroxime and ciprofloxacin.

Fewer female patients were included in P1 than in P2, which does not completely exclude possible changes in antibiotic prescribing due to gender differences. Aghdassi et al. described an increased risk for surgical site infections (SSI) in men for colon surgery, no differences for endoscopic cholecystectomy/appendectomy, and an increased risk in women for hernia repair [27]. Other studies described differing results regarding gender-specific risk for developing SSI in abdominal surgery [28,29]. Furthermore, this study did not focus primarily on SSI, but on intra-abdominal infections, which leads us to conclude that the influence of the different gender distribution in both study periods had a negligible impact on the results regarding antibiotic prescribing.

There are some limitations to our study. The patient collectives might not be comparable to those of smaller hospitals, in terms of the extent of the surgical procedures. In addition, there was no control group. However, the “cross-over” design between W3 and W4 generated an internal control to demonstrate the effects of the AS ward rounds. We were able to show a sustained effect of AS interventions, in terms of overall antibiotic consumption for three months after their completion. How long the effect of interventions persists warrants further research.

Since local treatment guidelines for IAI were not available during the study periods, a combination of AS ward rounds and internal guidelines on antibiotic therapy in IAI could further improve prescribing in the future. In particular, it could help junior faculty to make their decisions based on these local treatment guidelines when AS consultation is not available. Furthermore, AS ward rounds with senior faculty members present could promote rapid and safe decisions on antibiotic therapy.

## 4. Materials and Methods

### 4.1. Study Setting and Population

This monocentric, retrospective observational study was conducted at the visceral surgery department of the university hospital, LMU Munich. Two independent prescribing analyses of three months each (18 May–18 July (P1); 19 September–19 November (P2)) were carried out on three general and one intermediate-care (IMC) wards of the department.

**Figure 3:**
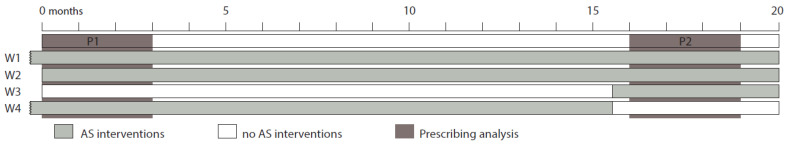
Study timeline of AS intervention and prescribing analysis for the four wards.

To investigate the impact (wards 1, 2, 3) and short-term sustainability (ward 4) of AS interventions, the two periods were chosen according to the provision of AS ward rounds. On ward 1 (W1, IMC), ward 2 (W2), and ward 4 (W4), AS interventions were introduced before P1. On W1 and W2, they were continued in P2, whereas the AS intervention was stopped after P1 on W4, and started only after P1 on ward 3 (W3) (Figure 3). All patients, aged 18 years or older, who received systemic antibiotics whilst on one of the four designated wards, were included in the study. Patients prescribed prophylactic antibiotics (perioperative or long-term prophylaxis), or with an incomplete set of data, were excluded. In addition, patients who stayed on the ward for more than 100 days were excluded, due to a possible bias of the results, as the hospital stay distinctly exceeded the observation period of three months. The study was approved by the ethics committee of the university hospital, LMU Munich (19-906).

### 4.2. Intervention

A multidisciplinary ASP was introduced at the university hospital, LMU Munich in 2017. The AS team was composed of an infectious disease (ID) physician, an ID pharmacist, and a clinical microbiologist. Weekly ward rounds based on an audit and feedback strategy, in collaboration with the current surgeon on duty, started on W2 with the observation period (P1), and on W1 and W4 a few months earlier (Figure 3).

### 4.3. Data Collection and Definitions

All demographic and clinical data, choice of antibiotic agent, days of antibiotic therapy, and microbiological results were extracted manually from electronic patient records and irreversibly anonymized during data collection.

Diagnoses requiring hospitalization and the resulting surgeries were recorded and allocated to the organ concerned (bowel, liver, pancreas, skin, and soft tissue).

In the study, antibiotic consumption was measured in days of therapy per 100 patient days. Length of antibiotic therapy (LOT) was defined as in-hospital days of antibiotic therapy. Continuous days of antibiotic treatment were defined as one course of antibiotics (COA). After discontinuation of antibiotic treatment for more than one day, a new COA was determined, and several COAs could, therefore, be assigned to one patient.

To evaluate changes in antibiotic therapy, antibiotic agents were ranked according to their spectrum of activity against drug-resistant bacteria [30,31] (Table 3). Changes in prescribed antibiotics within one COA were separated according to de-escalation or escalation [30,31,32]. De-escalation was defined as a change of one or more antibiotics to an agent with a lower rank, or termination of one or more antibiotics in a combination therapy. If a restricted drug (e.g., linezolid, daptomycin, or tigecycline) was still included after modification of antibiotic therapy, the change was not classified as a de-escalation. Escalation was defined as changing the antibiotic therapy to an agent with a higher rank, or adding one or more antibiotics for additional coverage. IV-to-oral switch was defined as a conversion from intravenous to oral antibiotic therapy. Each COA was checked retrospectively for changes in antibiotic therapy, and evaluated by an ID physician and a pharmacist. Duration to iv-to-oral switch or de-escalation was measured as days between the beginning of the antibiotic treatment and the iv-to-oral switch/de-escalation. COAs with more than one iv-to-oral switch/de-escalation were excluded from this statistical analysis.

### 4.4. Outcomes and Data Analysis

To assess the impact of weekly AS ward rounds, overall antibiotic consumption, as well as antibiotic consumption of single substance classes and change in antibiotic therapy, were analyzed in this study. In-hospital mortality and readmission caused by an infection after 30 days were compared to ensure patient outcome was not negatively affected by the AS intervention. Short-term sustainability was defined as maintained positive effects of AS ward rounds on antibiotic prescribing in the following three months after stopping them.

A sample size calculation was performed for the outcome of changes in length of antibiotic therapy (LOT). With a targeted power of 0.8 and a low expected effect, it was calculated that 325 patients were required in each study arm. By including 299 patients within three months for P1, we were close to the intended study sample size. For P2, including 279 patients resulted in the according study period of 3 months.

All categorical variables are shown as numbers with frequencies. Continuous variables are presented according to the distribution as a mean with a standard deviation or a median with a range. Variation between the two study periods for categorial variables was tested using the χ2-test or Fisher´s exact test, and for continuous variables, the t-test or Mann-Whitney U test was used. Statistical significance was set at *p* < 0.05. The statistical analysis was performed with IBM SPSS Statistics 26.

## 5. Conclusions

Regular AS ward rounds most likely improved antibiotic use in the setting of visceral surgery by reducing antibiotic consumption and increasing de-escalation rates of antibiotic courses. However, AS ward rounds need to be performed continuously, as sustained effects were only observed regarding the overall antibiotic use.

## Figures and Tables

**Table 1:** Patient characteristics and descriptive data for all patients included, comparing P1 and P2.

Characteristics	P1 (*n* = 299)	P2 (*n* = 275)	*p*-Value
Age—years, median (range)	62 (18–96)	62 (20–98)	0.809
Sex, female—no. (%)	110 (36.8)	126 (45.8)	0.028
Charlson comorbidity index—median (range)	2 (0–8)	2 (0–10)	0.637
Penicillin allergy—no. (%)	23 (7.7)	19 (6.9)	0.719
Diagnosis—no. (%)			0.948
Bowel	127 (42.5)	112 (40.7)	0.671
Liver	61 (20.4)	53 (19,3)	0.735
Pancreas	29 (9.7)	31 (11,3)	0.538
Skin and soft tissue	22 (7.4)	23 (8.4)	0.654
Others	60 (20.1)	56 (20.4)	0.930
No surgery	87 (29.1)	62 (22.5)	0.074
ICU—no. (%)	31 (10.4)	35 (12.7)	0.376
Length of hospital stay—days, median (range)	14 (2–90)	15 (2–79)	0.677
In-hospital mortality—no. (%)	3 (1.0)	2 (0.7)	0.722
Rate of readmission for infection after 30 days—no. (%)	25 (8.4)	20 (7.3)	0.621
Length of therapy (LOT)—days, median (range)	8 (1–73)	8 (1–77)	0.814
LOT < 5 d—no. (%)	50 (16.7)	45 (16.4)	0.908
LOT 5–9 d—no. (%)	121 (40.5)	108 (39.3)	0.770
LOT 10–14 d—no. (%)	47 (15.7)	55 (20.0)	0.180
LOT ≥ 15 d—no. (%)	81 (27.1)	67 (24.4)	0.456
Multidrug-resistant bacteria			
gram-positive	6	6	0.884
gram-negative	9	10	0.675

**Table 2:** Changes in antibiotic therapy for all courses of antibiotics, comparing P1 and P2.

	W1/2			W3			W4		
	P1 ^a^	P2 ^a^	*p*-Value	P1	P2 ^a^	*p*-Value	P1 ^a^	P2	*p*-Value
Courses of antibiotics	113	90		130	120		99	102	
Route of antibiotics at beginning of therapy—no. (%)			0.425			0.649			0.941
Intravenous	99 (87.6)	82 (91.1)		117 (90.0)	110 (91.7)		88 (88.9)	91 (89.2)	
Oral	14 (12.4)	8 (8.9)		13 (10.0)	10 (8.3)		11 (11.1)	11 (10.8)	
iv-to-oral switch	42 (42.4)	36 (43.9)	0.842	41 (35.0)	37 (33.6)	0.824	26 (29.5)	19 (20.9)	0.181
De-escalation	29 (25.7)	36 (40.0)	0.030	20 (15.4)	29 (24.2)	0.081	20 (20.2)	16 (15.7)	0.404
Escalation	43 (38.1)	32 (35.6)	0.714	35 (26.9)	22 (18.3)	0.106	25 (25.3)	19 (18.6)	0.256
	*n* = 41 ^b^	*n* = 35 ^b^		*n* = 40 ^b^	*n* = 36 ^b^		*n* = 24 ^b^	*n* = 17 ^b^	
Duration to iv-to-oral switch—days, median (range)	6 (1–21)	6 (1–15)	0.757	5 (1–20)	6.5 (1–20)	0.722	5 (2–13)	4 (2–28)	0.649
	*n* = 28 ^b^	*n* = 33 ^b^		*n* = 20 ^b^	*n* = 28 ^b^		*n* = 18 ^b^	*n* = 13 ^b^	
Duration to de-escalation—days, median (range)	6 (1–21)	6 (1–15)	0.738	5.5 (1–29)	6.5 (2–20)	0.674	6.5 (1–16)	5 (2–21)	0.809

^a^ AS Intervention, ^b^ Courses of antibiotics with more than one iv-to-oral switch/de-escalation were excluded from this statistical analysis.

**Table 3:** Antibiotic ranking [30].

Rank 1	narrow spectrum penicillins, first- and second-generation cephalosporins, co-trimoxazole, doxycycline, oral fosfomycin, metronidazole
Rank 2	aminopenicillin/beta-lactamase inhibitor, third-generation cephalosporins, fluoroquinolones, macrolides, clindamycin
Rank 3	fourth-generation cephalosporines, carbapenems, piperacillin/tazobactam, vancomycin
Rank 4	daptomycin, linezolid, tigecycline

## Data Availability

The data presented in this study are available on request from the corresponding author.

## Supplementary Materials

The following are available online at https://www.mdpi.com/article/10.3390/antibiotics10121518/s1, Table S1: Antibiotic consumption for single substances for the four study wards, comparing P1 and P2.
