# Supplementary material for: Impact and Sustainability of Antibiotic Stewardship on Antibiotic Prescribing in Visceral Surgery
Source: Antibiotics (Basel). 2021 Dec 11;10(12):1518. doi: 10.3390/antibiotics10121518 (PMC8698864; doi:10.3390/antibiotics10121518)
Supplement: Supplementary file 1 [file antibiotics-10-01518-s001.zip › antibiotics-1482618-supplementary.pdf]

Table S1 Antibiotic consumption for single substances for the four study wards comparing P1 and P2

	W1 - P1 [DOT/100PD]	W1 - P2 [DOT/100PD]	W2 - P1 [DOT/100PD]	W2 - P2 [DOT/100PD]	W3 - P1 [DOT/100PD]	W3 -P2 [DOT/100PD]	W4 - P1 [DOT/100PD]	W4 - P2 [DOT/100PD]
amoxicillin	0.9	0.0	0.2	0.0	0.4	0.0	0.4	0.0
amoxicillin/clavulanic acid	0.0	0.0	4.0	3.7	3.9	2.6	2.9	1.2
ampicillin	0.0	0.2	0.0	0.1	0.0	0.0	0.0	0.1
ampicillin/sulbactam	0.0	0.0	0.4	0.2	0.7	0.9	0.1	0.1
azithromycin	0.0	0.0	0.0	0.0	0.0	0.2	0.0	0.0
cefazolin	0.0	0.0	1.4	0.0	0.0	0.1	1.0	0.0
cefepime	3.8	1.6	1.1	0.0	0.0	1.2	0.5	1.6
cefpodoxime	0.0	0.0	0.0	0.0	0.0	0.8	0.0	0.0
ceftriaxone	2.2	0.4	2.7	2.5	4.2	0.5	2.7	2.0
cefuroxime	4.2	0.0	3.9	0.6	3.0	2.5	2.0	0.2
ciprofloxacin	5.5	3.2	13.6	10.2	10.5	4.9	9.5	7.9
clarithromycin	1.6	0.0	0.5	0.0	0.4	0.4	0.5	0.0
clindamycin	1.1	0.4	4.4	1.0	2.0	1.9	1.3	1.1
co-trimoxazole	0.0	3.0	0.6	1.8	0.5	2.1	0.2	2.7
daptomycin	0.0	0.0	0.0	0.2	0.0	0.0	0.0	0.0
doxycycline	0.0	0.0	0.0	0.0	0.0	0.5	0.0	0.0
flucloxacillin	0.4	0.4	0.0	0.0	0.4	0.2	0.0	0.0
fosfomycin	0.0	0.0	0.1	0.3	0.1	0.0	0.4	0.1
levofloxacin	0.0	0.0	0.0	0.0	0.0	0.0	0.0	0.1
linezolid	13.3	12.7	10.9	8.6	9.4	8.0	6.4	12.1
meropenem	23.5	22.2	14.2	11.5	9.8	9.8	10.8	13.2
metronidazole	9.5	3.6	11.1	6.5	12.8	5.5	8.0	4.3
moxifloxacin	1.5	0.6	3.2	3.1	0.5	1.7	2.0	2.0
penicillin G	0.0	0.0	1.4	0.0	0.4	2.4	1.3	0.2
penicillin V	0.0	0.0	0.0	0.0	0.0	0.4	0.2	0.0
piperazillin/tazobactam	29.1	30.1	19.9	16.1	21.5	21.4	18.5	24.5
tigecycline	1.5	1.6	1.6	0.0	0.2	0.6	0.8	0.6
vancomycin	11.8	1.4	0.8	1.9	2.9	0.7	1.7	0.4
overall	109.6	81.6	96.1	68.3	83.6	69.2	71.3	74.4
